# Citral modulates human monocyte responses to *Staphylococcus aureus* infection

**DOI:** 10.1038/s41598-021-01536-4

**Published:** 2021-11-11

**Authors:** Hellen Braga Martins Oliveira, Nathan das Neves Selis, Thamara Louisy Santos Brito, Beatriz Almeida Sampaio, Rafaela de Souza Bittencourt, Caline Novais Teixeira Oliveira, Manoel Neres Santos Júnior, Carolline Florentino Almeida, Palloma Porto Almeida, Guilherme Barreto Campos, Aline Teixeira Amorim, Jorge Timenetsky, Carla Cristina Romano, Ana Paula Trovatti Uetanabaro, Regiane Yatsuda, Lucas Miranda Marques

**Affiliations:** 1 Universidade Estadual de Santa Cruz, Rod. Jorge Amado, Km a6, Salobrinho, Ilhéus, Bahia 55662-900 Brazil; 2 Instituto Multidisciplinar em Saúde, Universidade Federal da Bahia, Rua Hormindo Barros, 58, Candeias, Vitória da Conquista, Bahia 45029-094 Brazil; 3 Departamento de Biologia Geral, Universidade Federal da Viçosa, Av. Peter Henry Rolfs s/n, Campus Universitário, Viçosa, Minas Gerais CEP: 36570-000 Brazil; 4 Instituto de Ciências Biomédicas, Universidade de São Paulo, Avenida Professor Lineu Prestes, 2415, Butantã, São Paulo, 05508-900 Brazil

**Keywords:** Antimicrobials, Pathogens

## Abstract

*Staphylococcus aureus* is a Gram-positive bacterium that is considered an important human pathogen. Due to its virulence and ability to acquire mechanisms of resistance to antibiotics, the clinical severity of *S. aureus* infection is driven by inflammatory responses to the bacteria. Thus, the present study aimed to investigate the modulating role of citral in inflammation caused by *S. aureus* infection. For this, we used an isolate obtained from a nasal swab sample of a healthy child attending a day-care centre in Vitória da Conquista, Bahia, Brazil. The role of citral in modulating immunological factors against *S. aureus* infection was evaluated by isolating and cultivating human peripheral blood mononuclear cells. The monocytes were treated with 4%, 2%, and 1% citral before and after inoculation with *S. aureus*. The cells were analysed by immunophenotyping of monocyte cell surface molecules (CD54, CD282, CD80, HLA-DR, and CD86) and cytokine dosage (IL-1β, IL-6, IL-10, IL-12p70, IL-23, IFN-γ, TGF-β, and TNF-α), and evaluated for the expression of 84 genes related to innate and adaptive immune system responses. GraphPad Prism software and variables with *P* values < 0.05, were used for statistical analysis. Our data demonstrated citral’s action on the expression of surface markers involved in recognition, presentation, and migration, such as CD14, CD54, and CD80, in global negative regulation of inflammation with inhibitory effects on NF-κB, JNK/p38, and IFN pathways. Consequently, IL-1β, IL-6, IL-12p70, IL-23, IFN-γ, and TNF-α cytokine expression was reduced in groups treated with citral and groups treated with citral at 4%, 2%, and 1% and infected, and levels of anti-inflammatory cytokines such as IL-10 were increased. Furthermore, citral could be used as a supporting anti-inflammatory agent against infections caused by *S. aureus*. There are no data correlating citral, *S. aureus*, and the markers analysed here; thus, our study addresses this gap in the literature.

## Introduction

*Staphylococcus aureus* is a coccus-shaped, Gram-positive bacterium belonging to the group of pathogens referred to as ‘ESKAPE’ (*Enterococcus faecium, Staphylococcus aureus, Klebsiella pneumoniae, Acinetobacter baumannii, Pseudomonas aeruginosa*, and *Enterobacter* species). It is considered an important human pathogen that is linked to a wide range of clinical manifestations, including respiratory infections, septic arthritis, endocarditis, and toxic shock syndrome^1–3^. Due to their high virulence and ability to acquire mechanisms of resistance to antibiotics, strains of *S. aureus* are known as methicillin-resistant *S. aureus* (MRSA), and can be found in hospital environments (HA-MRSA) or in community settings (CA-MRSA)^4–8^.

Because of the decreasing efficiency of conventional antibiotics, efforts have been made to decipher the mechanisms of action of plant extracts and essential oils, since numerous phytomolecules have demonstrated antimicrobial, insecticidal, antioxidant, and immunomodulatory activities^9–11^. In this context, citral (3,7-dimethyl-2,6-octadienal), a monoterpenic aldehyde formed by mixing 2 isomeric compounds (neral and geranial), demonstrates antibacterial, antifungal, analgesic, antispasmodic, antiparasitic, and immunomodulatory action, in addition to combatting nerve disorders^12–14^. Citral can be found in plants such as melissa (*Melissa officinalis*), holy grass (*Cymbopogon citratus*), and verbena (*Verbena officinalis*), among others, and is a potential candidate against infection by *S. aureus* strains^12,15^.

Staphylococcal infections are usually associated with severe inflammatory responses, and this pathogen has emerged as an even greater therapeutic challenge. Monocytes are antigen-presenting cells, and their expression of different cell-surface markers plays important roles in the infectious process or in disease remission. Their activation is necessary for initiating modulation of immune responses through gene transcription related to different pathways, including nuclear factor kappa B (NF-κB), leading to the production and secretion of pro-inflammatory mediators and cytokines^16^. These phagocytic cells are necessary in many inflammatory diseases, including those caused by *S. aureus*, as they migrate to the sites of extravascular infection by binding to endothelial adhesion molecules in response to chemoattractant stimuli produced in the presence of pathogens^17,18^. The adequate production of inflammatory mediators aims to eliminate infection by microorganisms^19^.

Immunomodulators are agents that affect the immune system by regulating molecules, such as cytokines, hormones, and peptides, among others, and stimulating or inhibiting events involving immune responses^20^. It is known that phytochemicals generally act through different mechanisms than conventional antibiotics and can be useful in treating antibiotic-resistant bacteria^10^. In a previous study, we analysed the anti-inflammatory action of citral. A positive effect was observed in reducing levels of microorganisms, as well as a significant decrease in the levels of tumour necrosis factor α (TNF-α) in treated mice^15^. In view of the acquired mechanisms of resistance of these microorganisms to different antibiotics, the present study investigated the role of citral immunomodulation in inflammation by *S. aureus*.

## Results

### Immunophenotyping of cell surface molecules by flow cytometry

**Figure 1:**
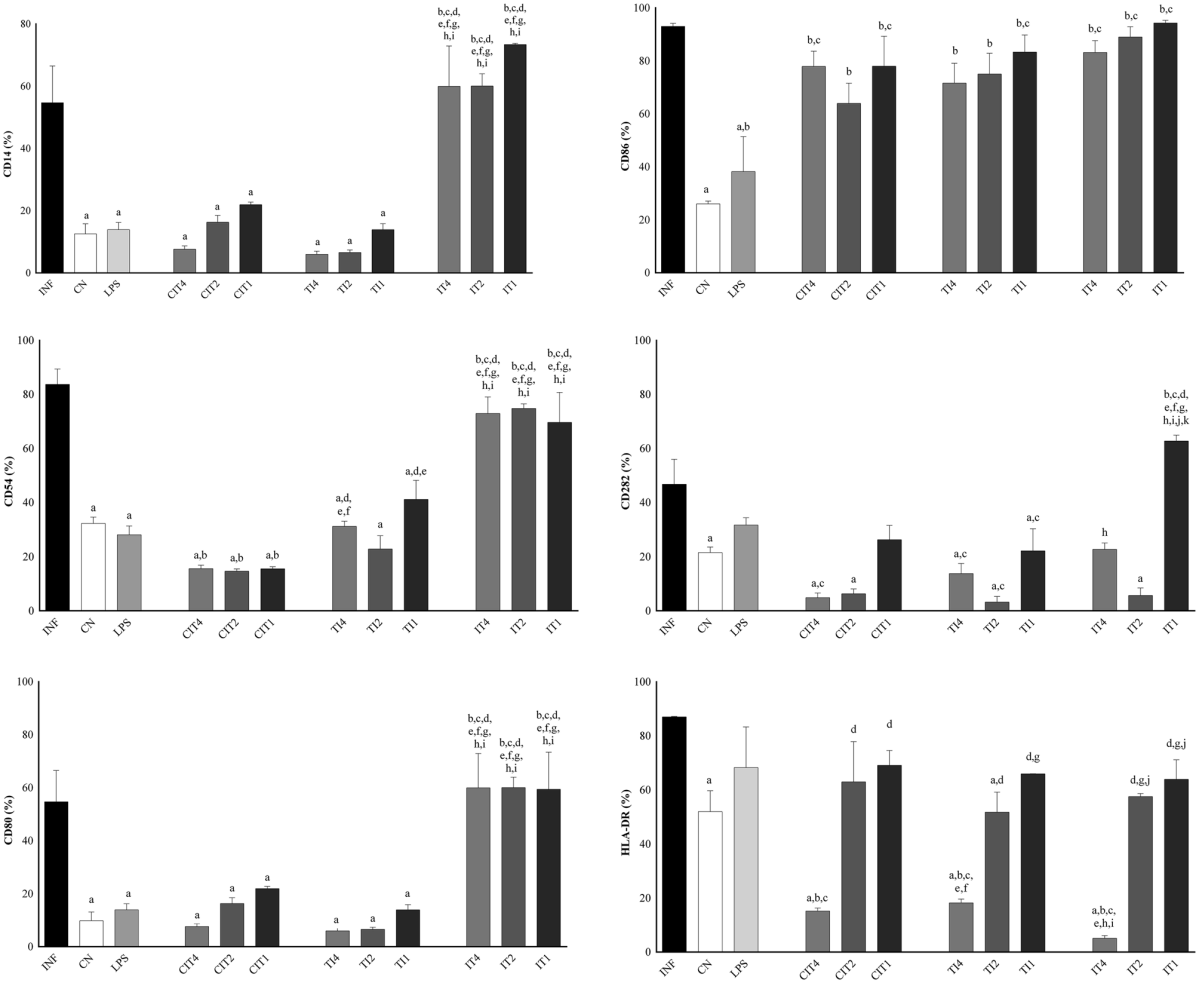
Immunophenotyping of cell surface molecules in monocytes (isolated from males) were performed by flow cytometry. Surface markers CD14, CD80, CD86, HLA-DR, CD82, and CD54. For immunophenotyping, monocytes were divided into the following groups: infected with isolate 80 (INF), negative control group (CN), groups with citral treatment (CIT) at 4%, 2%, and 1% (CIT4, CIT2, and CIT1), groups treated with citral at 4%, 2%, and 1% and infected (TI4, TI2, and TI1), and infected and treated groups with citral at 4%, 2%, and 1% (IT4, IT2, and IT1). Treatment for 30 min and infection for 6 h. Statistically significant differences (*P* < 0.05) compared to the INF control are indicated by letters: (**a**) compared to the negative control: (**b**) compared to LPS: (**c**) compared to CIT4: (**d**) compared to CIT2: (**e**) compared to CIT1: (**f**) compared to TI4: (**g**) compared to TI2: (**h**) compared to TI1: (**i**) compared to IT4: (**j**) compared to IT2: (**k**) Statistical significance (one-way ANOVA, with multiple comparisons followed by Tukey’s test; GraphPad Prism v.6.0).

Significant differences were observed between the groups for the markers CD14, CD54, CD80, CD86, CD282, and HLA-DR (Fig. 1). For CD14, CD54, and CD80, there were significant differences in their expression in the INF (infected with isolate 80) group compared to the CN (negative control), lipopolysaccharides (LPS), CIT (citral treatment), and TI (treated with 4%, 2%, and 1% citral and infected) groups. In addition, significant changes were observed between CN and LPS groups compared to INF group in terms of the expression levels of CD86, CN, CIT2, CIT1, and TI groups at three concentrations for CD282, and CN, CIT4, TI4, TI2, and IT4 (infected and treated with 4% citral) groups for HLA-DR. We observed a significant difference in the expression levels of CD54 and CD80 in the CN group compared to the CIT and IT groups at the three concentrations, and among the LPS, CIT, IT, and TI groups for CD86 compared to the CN group at the same three concentrations. CD282 showed a significant increase compared to the IT1 group, and HLA-DR compared to the CIT4, TI4, and IT4 groups. LPS was significant compared to most IT study groups involving the markers. CIT groups at the three concentrations showed a significant difference in the levels of CD14, CD80, and CD282 compared to the CN group, CD54 compared to the INF and CN groups, and CD 86 compared to the CN and LPS groups. We observed differences between treatments using 4%, 2%, or 1% citral only for HLA-DR. The results of the TI groups showed significant differences with CD14, CD80, and CD282 compared to the CN group. In addition, significant changes were observed for CD86 among the LPS TI4, and TI1 groups, and in CIT4 group for CD54, and TI2 and TI1groups for HLA-DR. The IT groups showed a significant difference in the number of monocytes compared to the INF, CN, LPS, CIT, and TI groups at the three concentrations for CD14, CD54, and CD80. We observed a significant difference in the CN and LPS groups for CD86. IT1 was significantly different in CD282 compared with CIT and TI at all concentrations and IT4 and IT2. Analysing HLA-DR, we observed that the IT2 and IT1 groups revealed significant differences compared to the CIT4, TI4, and IT4 groups. For the markers CD14 and CD80, both dose-dependent and CD54, citral showed a prophylactic and anti-inflammatory profile, being statistically significant in the treated and infected groups. This reduction was not observed in the infected and treated groups.

### Cytokine dosage

**Figure 2:**
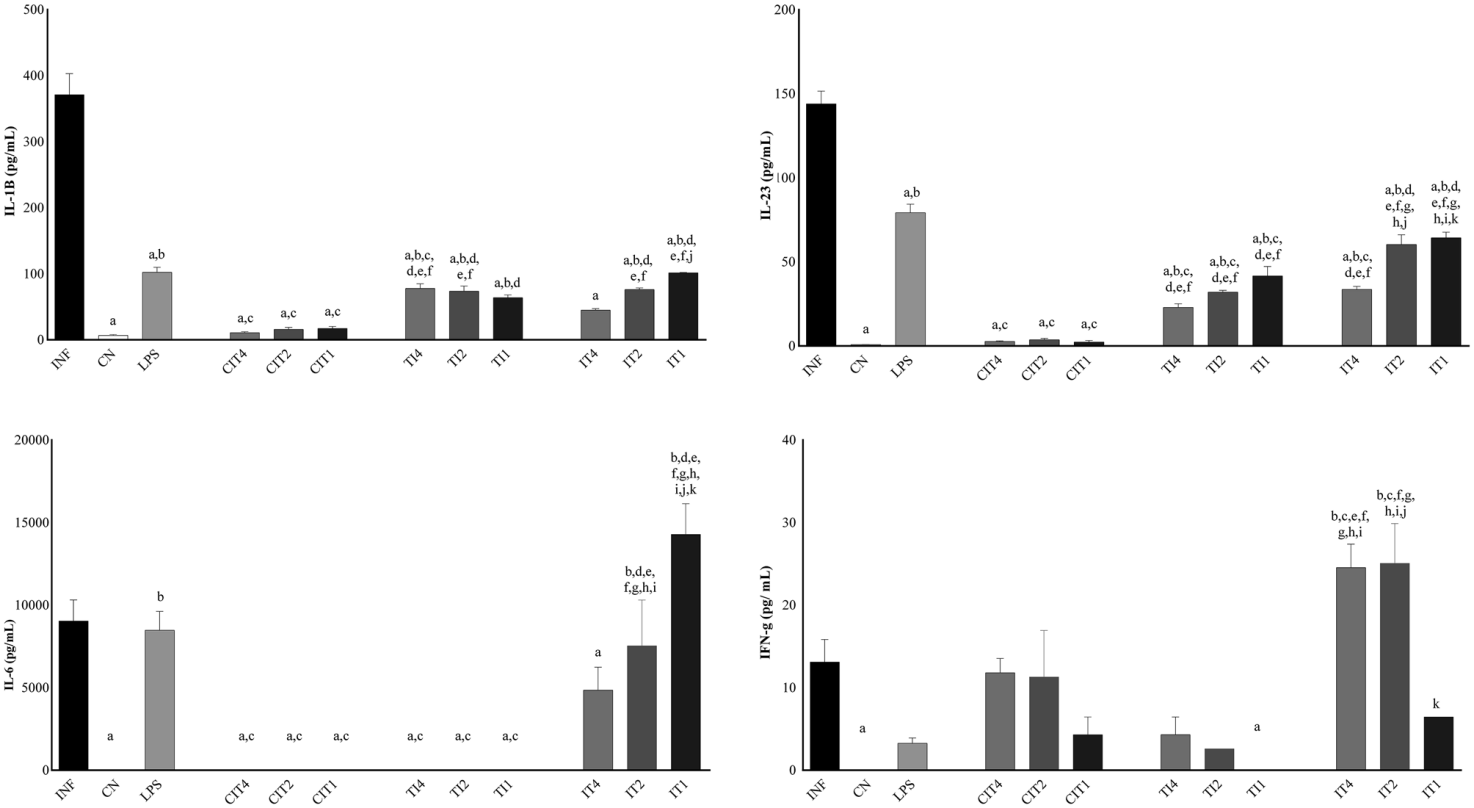
For analysis of cytokines IL-1β, IL-6, IL-23, and IFN-γ in human monocytes, the following groups were established: infected with isolate 80 (INF), negative control group (CN), groups treated with citral (CIT) at 4%, 2%, and 1% (CIT4, CIT2, and CIT1), groups treated with citral at 4%, 2%, and 1% and infected (TI4, TI2, and TI1), and infected and treated groups with citral at 4%, 2%, and 1% (IT4, IT2, and IT1). Treatment for 30 min and infection for 6 h. Statistically significant differences (*P* < 0.05) compared to the INF control are indicated by letters: (**a**) compared to the negative control: (**b**) compared to LPS: (**c**) compared to CIT4: (**d**) compared to CIT2: (**e**) compared to CIT1: (**f**) compared to TI4: (**g**) compared to TI2: (**h**) compared to TI1: (**i**) compared to IT4: (**j**) compared to IT2: (**k**) Statistical significance (Mann–Whitney one-tailed; GraphPad Prism, v.6.0).

**Figure 3:**
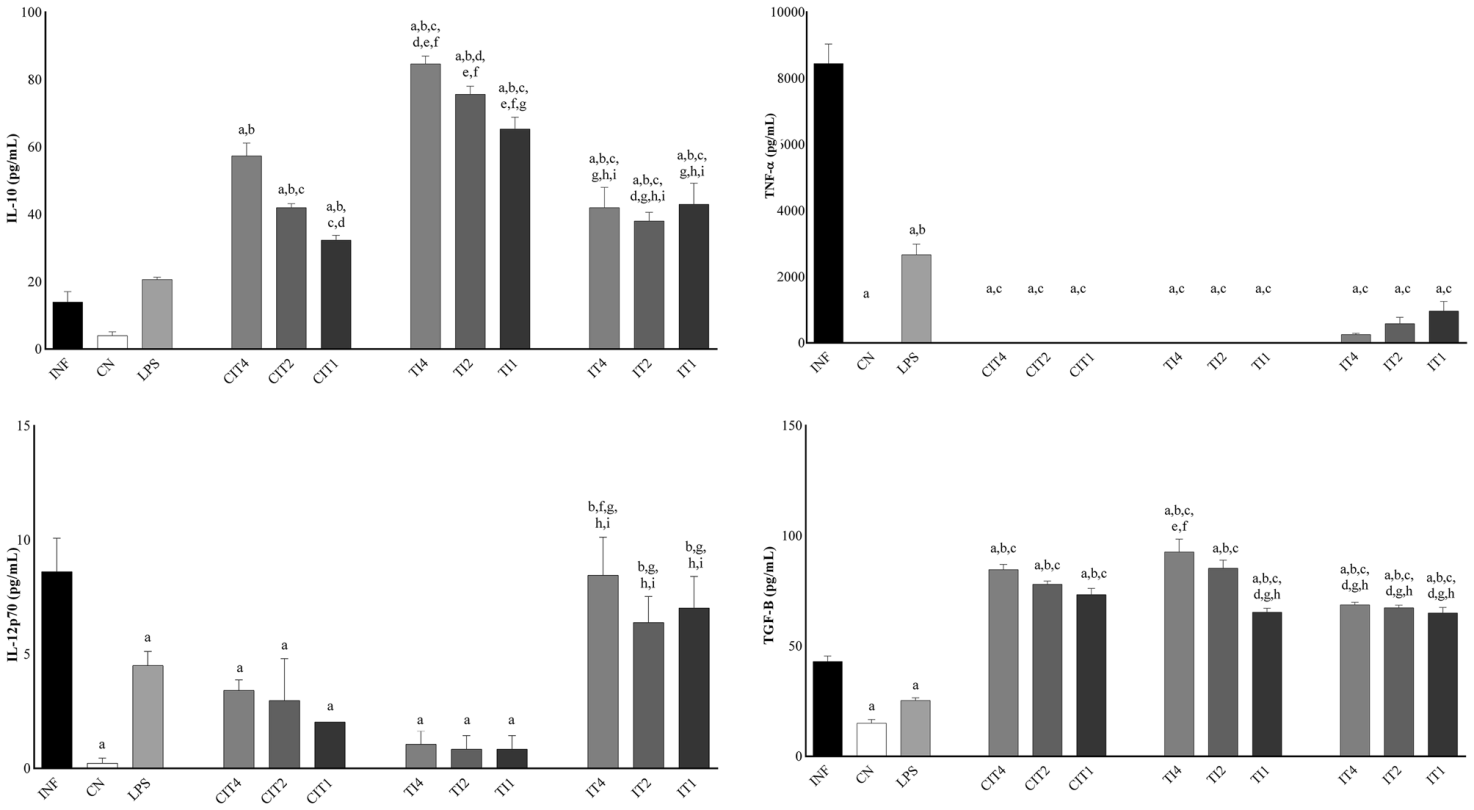
For analysis of cytokines IL-10, IL-12p70, TGF-β, and TNF-α in human monocytes, the following groups were established: infected with isolate 80 (INF), negative control group (CN), groups with citral treatment (CIT) at 4%, 2%, and 1% (CIT4, CIT2, and CIT1), groups treated with citral at 4%, 2%, and 1% and infected (TI4, TI2, and TI1), and infected and treated groups with citral at 4%, 2%, and 1% (IT4, IT2, and IT1). Treatment for 30 min and infection for 6 h. Statistically significant differences (*P* < 0.05) compared to the INF control are indicated by letters: (**a**) compared to the negative control: (**b**) compared to LPS: (**c**) compared to CIT4: (**d**) compared to CIT2: (**e**) compared to CIT1: (**f**) compared to TI4: (**g**) compared to TI2: (**h**) compared to TI1: (**i**) compared to IT4: (**j**) Statistical significance (Mann–Whitney One-tailed; GraphPad Prism, v.6.0).

By analysing cytokines in human monocytes, levels of interleukin (IL)-1β, IL-23, and TNF-α were found to be decreased, and the levels of IL-10 and transforming growth factor (TGF)-β were increased when the CN, LPS, CIT, TI, and IT groups at all concentrations were compared with the INF (infected with isolate 80) group. IL-12p70 and IL-6 levels were significantly decreased in the CN, LPS, CIT, and TI groups at all concentrations compared to the INF group (Figs. 2, 3). Regarding the CN group, IL-1β, IL-6, IL-23, and TNF-α showed a significant difference in their expression levels compared to the LPS group, IL-10 and TGF-β with CIT groups at the three concentrations, IL-1β, IL-10, IL-23, and TGF-β with TI groups at the three concentrations, and IL-12, IL-23 and TGF-β with IT groups at all concentrations. From the LPS results, we observed significant differences in the INF group in terms of the levels of IL-12p70, interferon gamma (IFN-γ), and TGF-β in the CN group for IL-6, and for INF and CN groups, the levels of IL-1β, IL-23, and TNF-α. It was observed that treatment with citral (CIT) at all concentrations significantly decreased the levels of cytokines IL-1β, IL-6, IL-12p70, IL-23, and TNF-α, and significantly increased the levels of cytokines IL-10 and TGF-β compared to the INF group, demonstrating potent immunomodulation by this oil. In the TI groups, it was observed that treatment with citral at different concentrations changed the levels of cytokines, being significant for cytokines IL-10 and IL-23 at the three concentrations, IL-1β at concentrations of 4% and 2%, IL-10 and TGF-β at 2% and 1%, and IL-1β and TGF-β at a concentration of 1%. For the IT groups, a significant difference was observed for IL-1β, IL-10, IL-12p70, IL-23, IFN-γ, and TGF-β compared to most of the other study groups.

### Gene expression analysis

**Figure 4:**
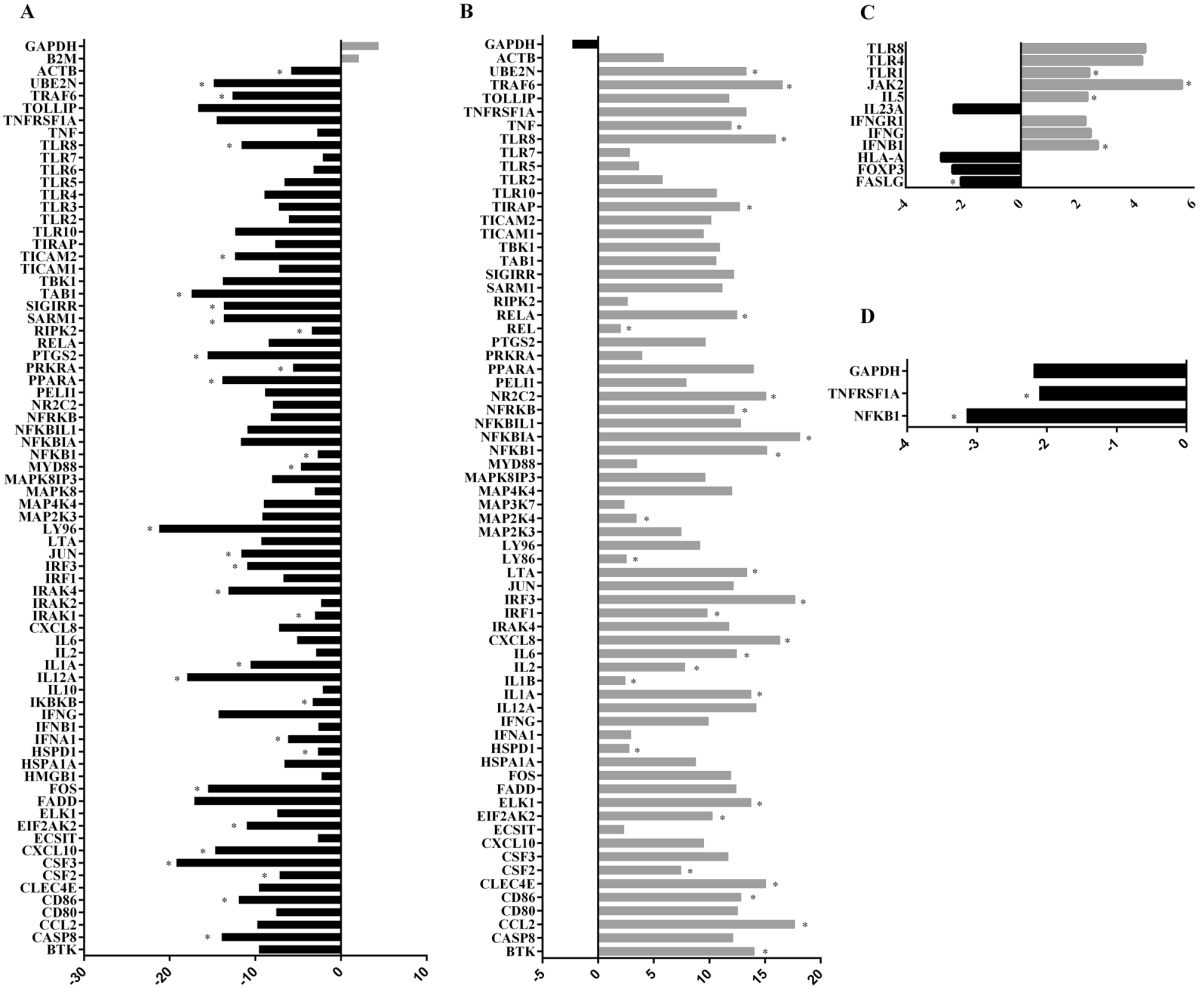
Gene expression analysis: up-regulated (grey) and down-regulated genes (black). The expression of genes was also evaluated in human monocyte samples of positive control groups (infected with *S. aureus*), negative control group, treated with citral group, and treated and infected group. (**A**) In the group infected with *S. aureus* compared with negative control group, there were statistical differences involving 29 up-regulated genes (*BTK, CCL2, CD86, CLEC4E, CSF2, EIF2AK2, ELK1, HSPD1, IL1A, IL1B, IL2, IL6, CXCL8, IRF1, IRF3, LTA, LY96, MAP2K4, NFKB1, NFKBIA, NFRKB, NR2C2, REL, RELA, TIRAP, TLR8, TNF, TRAF6,* and *UBE2N*; *P* < 0.05). (**B**) Seventy-four genes were regulated in the group treated and infected compared with the positive control groups. There were statistical differences between 31 down-regulated genes: *CASP8, CD86, CSF2, CSF3, CXCL10, EIF2AK2, FOS, HSPD1, IFNA1, IKBKB, IL12A, IL1A, IRAK1, IRAK4, IRF3, JUN, LY96, MYD88, NFKB1, PPARA, PRKRA, PTGS2, RIPK2, SARM1, SIGIRR, TBK1, TICAM2, TLR8, TRAF6, UBE2N,* and *ACTB*. (**C**) Twelve genes were regulated in the group treated and infected compared with the negative control group. There were statistical differences between 4 down-regulated genes: *FASLG, FOXP3, IL-23,* and *HLA-A*, and there were statistical differences between 9 up-regulated genes: *IFNB1, IFNG, IFNGR1, JAK2, TLR1, TLR4,* and *TLR8*. (**D**) Two genes were down-regulated in treated groups compared with the negative control group: *TNFRSF1A* and *NFKB1* (*P* < 0.05).

Gene expression was also evaluated in human monocyte samples from the following groups: infected group (INF), negative control group (CN), treated group (CIT), and treated and infected group (TI). Among the 84 genes analysed, 68 were regulated in groups infected with *S. aureus* compared with the CN group. There were statistical differences between the groups for 29 up-regulated genes related to toll-like receptor genes (*TLR8)*, response to bacterial pathogens *(CCL2, IL6, LTA, NFKB1)*, TLR signalling: *TICAM-*dependent *(MYD88-*independent*: IRF3, MAP2K4, TRAF6; MYD88*-dependent*: NR2C2, TIRAP, TLR8, TRAF6)*, downstream pathways and target genes: NFκB pathway (*BTK, LY96, NFKB1, NFKBIA, NFRKB, REL, RELA, TNF, UBE2N*), mammalian stress-activated protein kinases p38 and JNK (JNK/p38) pathway (*ELK1, MAP2K4, IL1B*), Janus kinases/signal transducer and activator of transcription (JAK/STAT) pathway (*CSF2, IL2, IL6*), interferon regulatory factor pathway *(IRF1, IRF3*), cytokine-mediated signalling pathway (*IL1A, IL1B, IL6, RELA*), regulation of adaptive immunity (*CD86, HSPD1, IL2, TRAF6),* adapters and proteins that interact with TLR (BTK, HSPD1, MAP2K4), and *EIF2AK2, UBE2N* (Fig. 4A).

Seventy-four genes were regulated in the treated and infected groups compared to the positive control groups. There were statistical differences between the groups for 31 down-regulated TLR genes (*TLR8*), response to bacterial pathogens (*FOS, IL12A, IL6, IRAK1, JUN, PTGS2*), TLR signalling: negative regulation (*SARM1, SIGIRR*), *TICAM-*dependent *(MYD88*-independent*: IRF3, TBK1, TRAF6, TICAM2*), *MYD88-*dependent (*TLR8, TRAF6*), downstream pathways and target genes: NFκB pathway (*CASP8, IKBKB, LY96, NFKB1, UBE2N*), JNK/p38 pathway (*FOS*), JAK/STAT pathway (*CSF2, CSF3*), interferon regulatory factor pathway (*IFNA1, IRF1, IRF3*), cytokine-mediated signalling pathway (*IL1A, IRAK1, SIGIRR*), regulation of adaptive immunity (*CD86, HSPD1, TRAF6*), adapters and proteins interacting with TLR (HSPD1, MYD88, RIPK2, SARM1, TICAM2), and EIF2AK2, IRAK1, IRAK4, PPARA, PRKRA, UBE2N (Fig. 4B).

Twelve genes were regulated in the treated and infected groups compared to the negative control group. There were statistical differences involving four down-regulated genes: *FASLG*, positive T regulatory cells (*FOXP3*), *IL-23*, and *HLA-A*, and there were statistical differences between nine upregulated genes: interferon regulatory factor pathway (*IFNB1, IFNG, IFNGR1*), *JAK2*, and regulated TLR genes (*TLR1, TLR4*, and *TLR8*) (Fig. 4C).

Two genes (response to bacterial pathogens *TNFRSF1A* and NFκB pathway (*NFKB1*)) were downregulated in the treated group compared with the CN group (Fig. 4D).

### Compilation of results

**Figure 5:**
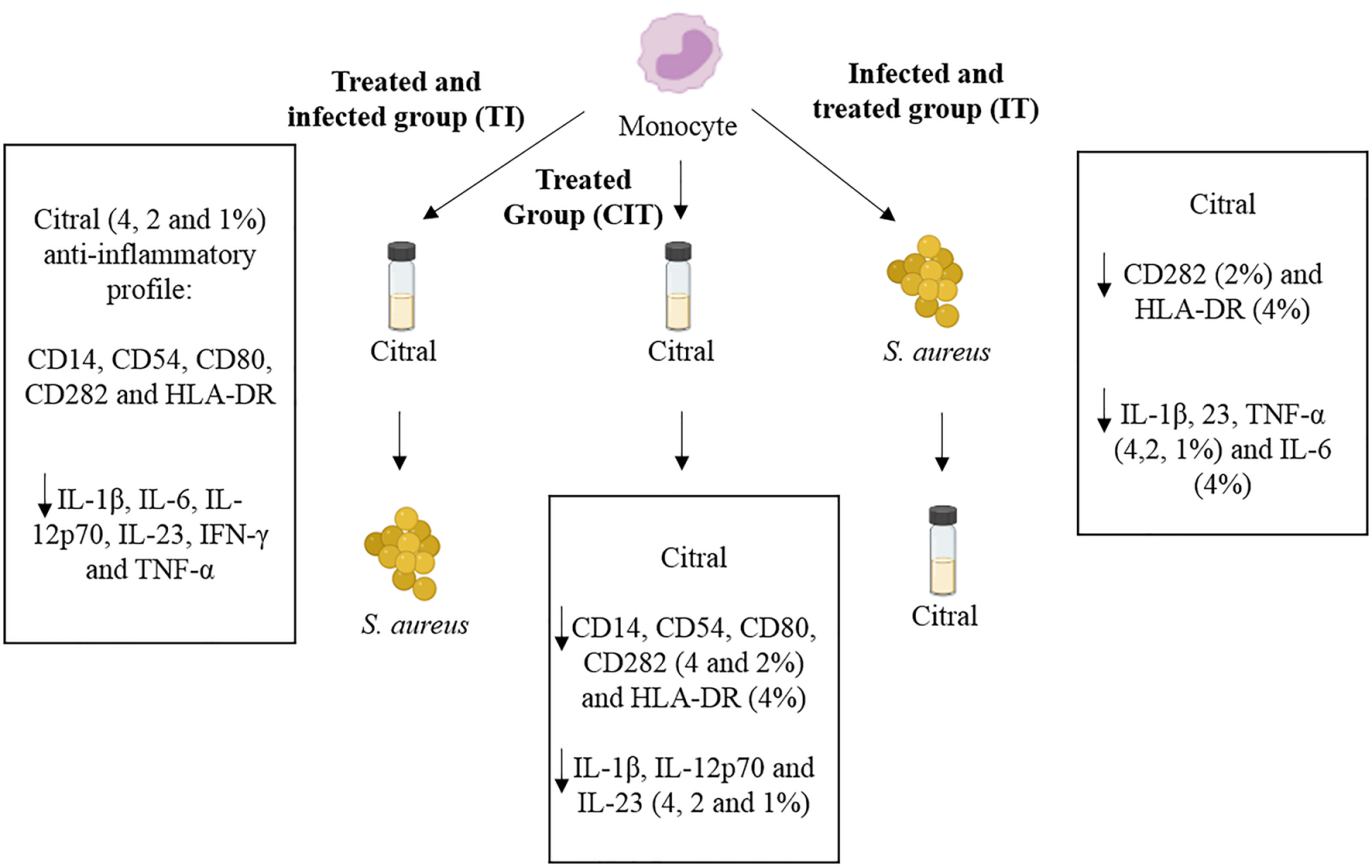
Proposed mechanism of action of citral in groups treated with citral and infected with *S. aureus* (TI), treated with citral (CIT), and infected with *S. aureus* and treated with citral (IT).

**Figure 6:**
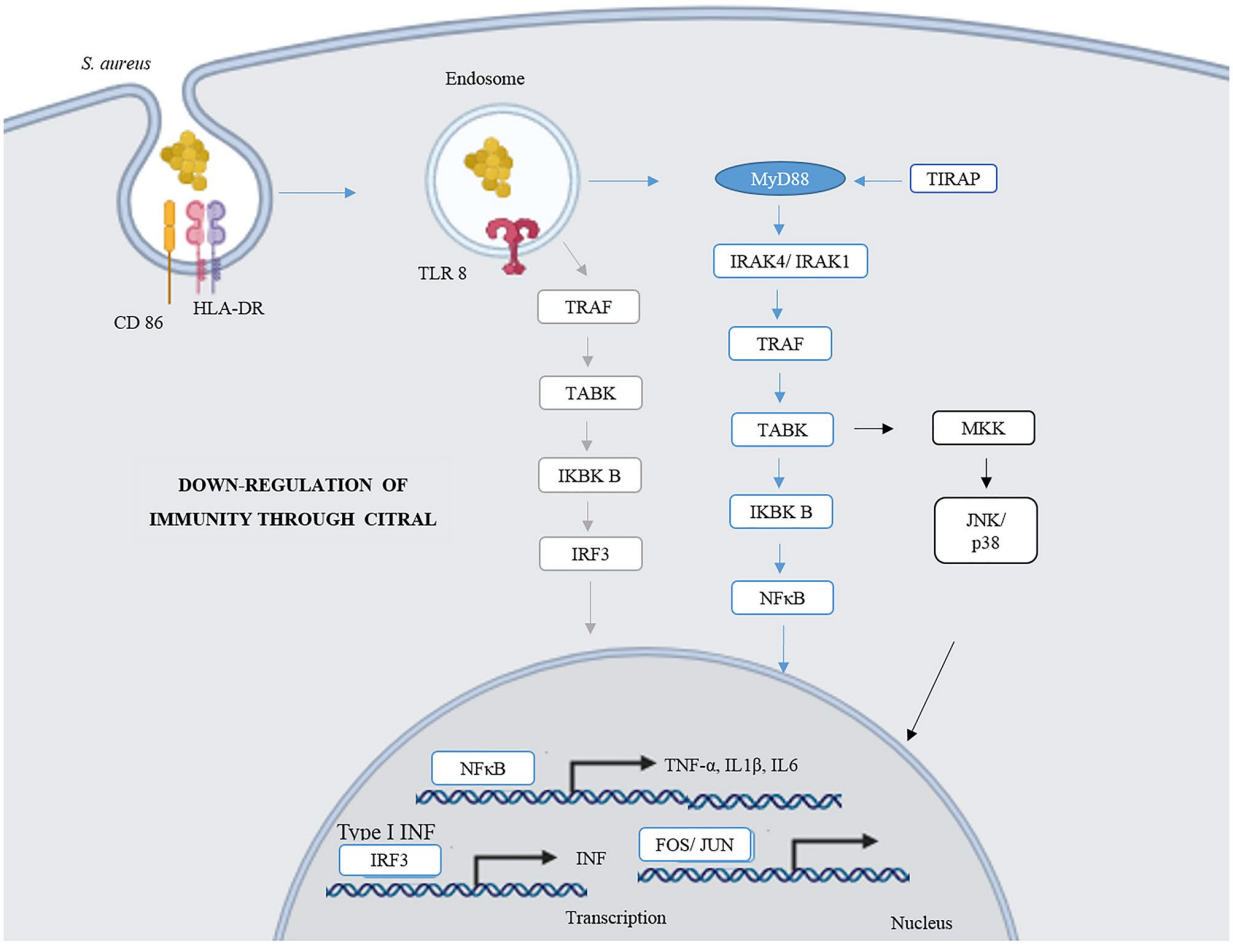
Citral provoked different intracellular responses in immune responses compared with those induced by *Staphylococcus aureus*. The study found actions on surface markers involved in recognition, presentation and migration, such as: CD14, CD54, CD80, CD282 and HLA-DR, global negative regulation of inflammation with inhibitory effects on NF-*κ*B, JNK/p38, and INF pathways, consequently reducing expression of cytokines IL-6, IL-12p70, and TNF-α (images: biorender.com).

In general, in the present study, citral demonstrated anti-inflammatory activity through its action on the cell-surface markers, such as CD14, CD54, CD80, CD282, and HLA-DR, and reduced the expression of cytokines IL-1β, IL-6, IL-12p70, IL-23, IFN-γ, and TNF-α in the TI (treated with citral at 4%, 2%, and 1% and infected) groups, and increased the levels of anti-inflammatory cytokines such as IL-10. In the IT (infected and treated with citral) group, citral demonstrated action on surface markers HLA-DR (4%) and CD282 (2%), and reduced expression of cytokines IL-1β, IL-23, and TNF-α in a dose-dependent manner at the three concentrations, and IL6 (4%) (Fig. 5). CIT inhibited the expression of surface markers CD14, CD54, CD80, CD282, and HLA-DR, and reduced expression of cytokines IL-1β, IL12p70, and IL-23. In addition, we observed that TLR8 is a sensor of the Gram-positive species *S. aureus*. When treated with citral, there was global negative regulation of inflammation-related genes, with down-regulation of the activation of NF-*κ*B, JNK/p38, and INF signalling pathways (Fig. 6).

## Discussion

There are no data in the literature concerning the modulation of citral, *S. aureus*, and monocyte markers measured by flow cytometry. Essential oils have been shown to possess a wide range of pharmacological properties and immunomodulatory activities, with promising results. When we compared CD282 expression in the INF group with the CIT or TI groups, we observed a significant reduction in the expression of this marker. There is evidence that TLR2 found in monocytes is involved in detecting *S. aureus* pathogen-associated molecular pattern pathway heterodimers, acting with other co-receptors in bacterial phagocytosis and inducing the synthesis of pro-inflammatory mediators^21,22^. The difference in the levels of CD54, which acts on the migration of leukocytes, was statistically significant between the IT and TI groups at all analysed concentrations. We observed that CD54 expression level was significantly reduced in the CN, LPS, CIT, and TI groups compared to that in the INF group. Another study observed positive regulation of different adhesion molecules such as VLA-4 (CD49d/CD29) and ICAM-1 (CD54) in pulmonary endothelium, leading to inflammatory changes in tissues^23^.

CD80, CD86, and HLA-DR are involved in antigen presentation and activation^24,25^. Elevated expression of this marker may occur due to the increased inflammatory processes involved in infection or treatment of these cells. In the expression of CD80, we observed differences between treatments using citral at 4%, 2%, or 1%, and CD80 expression level significantly increased in the different groups, depending on the dose, compared to the INF group. Another study employing a murine model of pneumonia caused by MRSA found that mice with CD80- and CD86-deficiency had significant reductions in pro-inflammatory cytokine levels and had significantly improved survival rates, corroborating the hypothesis that attenuation of inflammation in infectious conditions minimizes damage and promotes recovery^26^. A reduction in the expression of this molecule denotes a lower capacity of the adaptive immune system, and in this study, reduction in HLA-DR levels occurred when the cells were treated with 4% citral^27^. Other authors have demonstrated that reduction in HLA-DR receptor abundance leads to reduced activation of the adaptive immune system^25^.

Following this perspective, for CD14, we observed a potential immunosuppressive action of citral on cells involved in innate immunity, thus having an anti-inflammatory profile. The reduction promoted by citral on CD54 molecules proved to be positive in preventing hyperinflammation. Citral is a potential anti-inflammatory agent against *S. aureus* infections by reducing the expression of CD80 in treated group or when used prophylactically. CD86 levels are not changed due to compensatory mechanisms of the immune system, and further studies are needed to elucidate such patterns. It is possible that attenuation of the expression of CD282 molecules is not of interest because it stimulates inflammatory responses. In short, citral exhibited an anti-inflammatory profile in terms of CD14, CD54, and CD80 markers.

Another parameter evaluated to investigate inflammatory modulation by citral is its cytokine activity. Cytokines may mediate defence against bacterial infections by activating innate immune cells and shaping adaptive immune responses. With pathogen recognition by pattern recognition receptors (PRRs), signal transduction pathways trigger phosphorylation of STATs, JAKs, mitogen-activated protein kinases (MAPKs), or the translocation of the NF-κB. As a result, the production and release of pro-inflammatory cytokines, such as IFN-γ, TNF-α, IL-1, IL-6, IL-12, and IL-18^28,29^. M1 macrophages are induced by Th1 cytokines such as IFNγ, TNF-α, or LPS, and typically attack microorganisms and the majority of TLRs^30^. After infection, TLRs are activated, leading to an increase in the expression of molecules contributing to the activation of T cells, which are fundamental in responses by the adaptive immune system and for cytokine production^21^. M2 macrophages are induced by Th2 cytokines (IL-4, IL-13, IL-10, and TGF-β), and are characterised by phagocytosis and resolution of inflammation^31^.

While comparing gene expression in the different study groups, we observed the expression of genes related to activation of the inflammatory response pathway involving TLR8. TLR8 is an endosomal sensor of monocytes and macrophage RNA degradation products in human phagocytes, and are involved in the recognition of viral and bacterial pathogens^32,33^. Studies of these pathways have shown that essential oils from chamomile, lemongrass, and sandalwood also exhibit significant inhibition of TNF-α, which otherwise initiates inflammatory responses by the NF-κB signalling pathway^34^. Another study revealed a specific role of TLR8 in *S. aureus* infection of primary monocytes and monocyte-derived macrophages (MDM) via activation of TAK1-IkB kinase (IKKβ)-IFN regulatory factor 5 (IRF5) signalling pathway, which induces the production of IFN-β, TNF, and IL-12^35^. Other authors demonstrated in models of LPS-induced inflammation that treatment with dry ginger extract, composed of geranial and neral isomers, decreases the degradation of IκB-α complexes, resulting in lower release of NFκB, in addition to the phosphorylation of ERK1/2, SAPK/JNK, and p38 MAPKs^36^. Another study demonstrated that neral and geranial have inhibitory efficacy against inflammatory proteins such as IL-6, IL-8, TNF-α, and IκB expressed by macrophages after LPS stimulation^37^. Other authors observed that at a 66-μM dose, citral could significantly inhibit the expression of iNOS and IκB kinase (IKK) in RAW264.7 macrophages stimulated by LPS. They also observed a decrease in the phosphorylation of MAP kinases, such as p38 ERK1/2 and JNK, and a decrease in plasma levels of pro-inflammatory cytokines. Activation of the MyD88/TRAF6 cascade leads to the activation of NFκB and JNK, which are essential for producing pro-inflammatory cytokines^38^.

LPS activate macrophages, which can induce inflammatory cytokine production. When present in an organism, they are transferred by an LPS-binding protein to another protein (CD14) present on the surface of monocytes, macrophages, dendritic cells, and neutrophils, generating a signal and binding to the TLR-4 glycoprotein receptor, consequently leading to the production of pro-inflammatory cytokines such as IL-1, IL-6, IL-8, and TNF-α^39^. We observed an increase in the levels of all cytokines in monocytes infected with *S. aureus* compared with the negative control group, which was also observed in another study, showing induction of IL-1β, IL-6, and IL-12p70 in monocytes in vitro, tending towards a polarization of the Th1 immune response in vivo^40^. Other authors observed that staphylococcal exocellular lipoteichoic acid activates pro-inflammatory cytokines (TNF-α, IL-6, and IL-1) in a murine macrophage cell line^41^. In this study, we observed significantly reduced levels of IL‐1β, IL‐6, IL12p70, IL-23, and TNF‐α in the CIT and TI groups compared to the INF group. Citral (0.36 g kg^−1^) significantly reduced levels of cytokines IL‐1β, IL‐6, and TNF‐α in MRSA-infected mice, and restored the levels of cytokines in mice to within the normal range^42^.

In a previous study, our group analysed the anti-inflammatory action of citral in an air-pocket model in mice infected with *S. aureus* and untreated or treated with citral. A significant decrease was observed in the levels of TNF-α in mice treated with citral^15^. Consistent with the results observed in this study, lemongrass oil, a major component of which is citral and is a mixture of tautomers geranial (trans-citral) and neral (cis-citral), has been reported to inhibit the expression of inflammatory cytokines such as IL-1β and IL-6 in peritoneal macrophages^43^. The arachidonic acid pathway modulates the inflammatory process and cellular immune responses, inhibiting the phagocytic action of macrophages, activity of APCs, and production of pro-inflammatory cytokines such as IL-12, and inducing the synthesis of IL-10^44^. In this study, IL-10 caused downregulation of IL12p70, since IL-10 levels were increased in groups with treated or treated and infected compared to infected and treated group, suggesting a prophylactic modulating effect on M1 macrophages.

This is a pioneering study, which evaluated modulation by citral in *S. aureus* infection through the expression of surface molecules, cytokine dosage, and expression of innate and adaptive immune response genes in monocytes. Thus, we believe that citral is a good candidate for developing new drugs against *S. aureus*, but the potential of citral as an immunomodulatory and anti-inflammatory agent should be further explored.

## Materials and methods

### Microorganisms

The present study selected *S. aureus* strain 80, characterised as CA-MRSA, SCC*mec* (IVA), presenting virulence genes *spa* type t002, ST/CC 5/5, *icaA, icaD*, and capable of forming biofilms. The strains used were obtained during other studies after approval by the Ethics Committee of Research with Human Beings of the Multidisciplinary Health Institute campus Anísio Teixeira under CAAE no. 08731912.5.0000.5556 (nasal strains)^45^. All techniques and procedures were performed in accordance with the relevant guidelines and regulations. MRSA ATCC 33591 was obtained from a commercial source. Each sample was plated with mannitol salt agar and incubated at 37 °C for 24 h.

### Citral

In these experiments, citral was commercially provided by Sigma-Aldrich® (MO, USA). For test runs, citral was diluted in propylene glycol at concentrations of 4%, 2%, and 1%, as recommended in the literature^46–48^.

### Blood collection

Peripheral blood samples were collected from three healthy men of reproductive age > 18 years who signed an informed consent form. Sample collection was performed following human ethical precepts approved by the Ethics Committee of Research with Human Beings of the Multidisciplinary Health Institute from the Anísio Teixeira campus under CAAE no. 79845717.1.0000.5556. Exclusion criteria for research participants were as follows: history of allergies, cardiac disorders, hypo/hypertension, circulatory or renal disorders, diabetes, medical treatment with anti-inflammatory medication or antibiotics, and daily consumption of alcohol or a smoker. Before collection, participants were screened using a questionnaire, and their blood pressure was measured. To confirm good general health, 15 mL of blood was drawn to determine cell counts, blood glucose, total cholesterol, cholesterol-LDL, cholesterol-HDL, cholesterol-NON HDL, triglycerides, aspartate transaminase, and alanine aminotransferase after 12 h of fasting.

### Isolation and culture of human peripheral blood mononuclear cells (PBMCs)

Blood samples were centrifuged to remove the plasma, diluted in 1 × phosphate buffered saline (PBS), and centrifuged using a Histopaque density gradient (1.077 g/mL, Sigma-Aldrich). After centrifugation, mononuclear cells were transferred to a new centrifuge tube and resuspended in RPMI-1640 culture medium (Gibco BRL, Gaithersburg, MD, USA) supplemented with 10% bovine foetal serum (Gibco BRL). The cells were counted in a Neubauer chamber and viability was determined by trypan blue exclusion assay (Sigma-Aldrich). After counting, viable cells were added to culture dishes and incubated at 37 °C under 5% CO_2_. Experimental groups were established for the study as follows: infected group (INF): infected with *S. aureus* only (with 10 μL inoculum of 1–5 × 10^8^ CFU/mL); negative control group (CN): only cells; positive control group with inoculation of LPS; treated groups (CIT): 60 μL citral at 4%, 2%, and 1% only; treated and infected groups (TI): treatment for 30 min with three concentrations before inoculation with bacteria; and infected and treated groups (IT): Infection with the bacteria was conducted for 6 h and treatment for 30 min for the three concentrations. Immunophenotyping of cell surface molecules was performed for all groups^49^.

### Immunophenotyping of cell surface molecules by flow cytometry

The cells used for this analysis were monocytes and molecules chosen because of their importance in the detection and elimination of the pathogen *S. aureus*. Flow cytometry data were acquired using a BD Accuri C6 flow cytometer (BD Biosciences, San Diego, CA, USA) and analysed using FlowJo™ software (v.10.0.7, BD Biosciences, Ashland, OR, USA). Flow cytometry calibration was performed using an aliquot of cells without any labelled probe present. Monocytes were first identified based on a dot plot of brightfield area versus aspect ratio, confirmed by staining with anti-human CD14 (clone 63D3). A gate was established around the population containing putative single cells; other events were gated-out and therefore not analysed. The anti-human mAbs used for multicolor flow cytometry were as follows: CD54 (clone HA58), CD282 (TL2.1), CD80 (2D10), HLA-DR (L243), and CD86 (IT2.2). All anti-human mAbs used were purchased from BioLegend (San Diego, CA, USA). At least 5.000 events were recorded for each target population.

### Cytokine dosage

Cytokine dosage was assessed in human monocytes using Luminex multiplex assay technology with a range of detection of 5–2000 pg/mL. This method allows for the simultaneous measurement of several analytes in a single sample. Thus, these assays were based on the ProcartaPlex Human Essential TH1/TH2 panel (Invitrogen) to determine levels of interferon (IFN) γ, IL-1β, IL-6, IL-10, IL-12p70, IL-23, TNF-α, and TGF-β.

### Gene expression analysis

Gene expression in human monocytes was verified by RT-qPCR. The experimental groups were infected group (INF): infection with *S. aureus*; negative control group (CN), only cells; treated group (CIT), citral at 4%; treated and infected group (TI): 30 min citral treatment at 4% and 6 h of infection. The cDNA obtained was subjected to analysis using the Human Innate and Adaptive Immune Responses PCR Array kit (Qiagen-SABioscience) for measurement of the expression of 84 genes involved in host responses to bacterial infection (Supplementary Table 1). This array includes genes related to host defences against bacteria represented in this array, including genes involved in detecting bacteria and genes involved in the acute-phase response, complement activation, inflammatory responses, and antibacterial humoral responses. All procedures were performed according to the manufacturer’s instructions.

### Statistical analysis

GraphPad Prism v.6.0 (GraphPad Software, San Diego, CA, USA) was used for analysis. To analyse cytokine dosage, a nonparametric statistic was evaluated: one-tailed Mann–Whitney *U*-test. For flow cytometry, analyses were performed using one-way ANOVA with multiple comparisons followed by Tukey’s test. For gene expression, data were generated and analysed using the kit software, and GraphPad was used to graphically represent the results. Statistical differences were considered significant at *P* < 0.05 using a 95% confidence interval.

### Ethics declarations and approval for human experiments

Ethics approval and consent were deemed unnecessary in this study, according to the Animal Ethics Committee (AEC) of the Multidisciplinary Health Institute, Federal University of Bahia. The bacterial strain used was obtained from other studies after approval by the Ethics Committee of Research with Human Beings of the Multidisciplinary Health Institute campus Anísio Teixeira under CAAE nº08731912.5.0000.5556 (nasal strain). Informed consent was obtained from parents or guardians. All methods were performed in accordance with the relevant guidelines and regulations.

## Supplementary Information

Supplementary Information.

## Data Availability

The datasets used and/or analysed during the current study are available from the corresponding author upon reasonable request.

## Abbreviations

ACTB: Actin, beta
APCS: Amyloid P component, serum
B2M: Beta-2-microglobulin
C3: Complement component 3
CA-MRSA: Community-acquired methicillin-resistant *Staphylococcus aureus*
CASP1: Caspase 1, apoptosis-related cysteine peptidase (interleukin 1, beta, convertase)
CC: Clonal complex
CCL2: Chemokine (C–C motif) ligand 2
CCL5: Chemokine (C–C motif) ligand 5
CCR4: Chemokine (C–C motif) receptor 4
CCR5: Chemokine (C–C motif) receptor 5
CCR6: Chemokine (C–C motif) receptor 6
CCR8: Chemokine (C–C motif) receptor 8
CD14: CD14 molecule
CD282: CD282 molecule
CD4: CD4 molecule
CD40: CD40 molecule, TNF receptor superfamily member 5
CD40LG: CD40 ligand
CD54: CD54 molecule
CD80: CD80 molecule
CD86: CD86 molecule
CD8A: CD8a molecule
Cholesterol-HDL: High-density lipoprotein cholesterol
Cholesterol-LDL: Low-density lipoprotein cholesterol
CIT: Treated groups
CN: Negative control group
CRP: C-reactive protein, pentraxin-related
CSF2: Colony stimulating factor 2 (granulocyte–macrophage)
CXCL10: Chemokine (C–X–C motif) ligand 10
CXCL8: Interleukin 8
CXCR3: Chemokine (C–X–C motif) receptor 3
DDX58: DEAD (Asp-Glu-Ala-Asp) box polypeptide 58
ESKAPE: *Enterococcus faecium, S. aureus, Klebsiella pneumoniae, Acinetobacter baumannii, Pseudomonas aeruginosa* And Enterobacter species
FASLG: Fas ligand (TNF superfamily, member 6)
FOXP3: Forkhead box P3
GAPDH: Glyceraldehyde-3-phosphate dehydrogenase
GATA3: GATA binding protein 3
HA-MRSA: Hospital-acquired methicillin-resistant *Staphylococcus aureus*
HLA-A: Major histocompatibility complex, class I, A
HLA-E: Major histocompatibility complex, class I, E
HPRT1: Hypoxanthine phosphoribosyltransferase 1
*icaA*: Intercellular adhesion gene cluster type A
*icaD*: Intercellular adhesion gene cluster type A
ICAM1: Intercellular adhesion molecule 1
ICU: Intensive care unit
IFNA1: Interferon, alpha 1
IFNAR1: Interferon (alpha, beta and omega) receptor 1
IFNB1: Interferon, beta 1, fibroblast
IFNG: Interferon, gamma
IFNGR1: Interferon gamma receptor 1
IL10: Interleukin 10
IL-12p70: Interleukin 12
IL13: Interleukin 13
IL17A: Interleukin 17A
IL18: Interleukin 18 (interferon-gamma-inducing factor)
IL1A: Interleukin 1, alpha
IL1B/IL-1β: Interleukin 1, beta
IL1R1: Interleukin 1 receptor, type I
IL2: Interleukin 2
IL-23/IL23A: Interleukin 23, alpha subunit p19
IL4: Interleukin 4
IL5: Interleukin 5 (colony-stimulating factor, eosinophil)
IL-6/IL6: Interleukin 6 (interferon, beta 2)
INF: Infected group
IRAK1: Interleukin-1 receptor-associated kinase 1
IRF3: Interferon regulatory factor 3
IRF7: Interferon regulatory factor 7
IT: Infected and treated group
ITGAM: Integrin, alpha M (complement component 3 receptor 3 subunit)
JAK2: Janus kinase 2
JNK/p38: Mammalian stress-activated protein kinases p38 and JNK
LPS: Lipopolysaccharides
LY96: Lymphocyte antigen 96
LYZ: Lysozyme
MAPK1: Mitogen-activated protein kinase 1
MAPK8: Mitogen-activated protein kinase 8
MBL2: Mannose-binding lectin (protein C) 2, soluble
MPO: Myeloperoxidase
MRSA: Methicillin-resistant *Staphylococcus aureus*
MX1: Myxovirus (influenza virus) resistance 1, interferon-inducible protein p78 (mouse)
MYD88: Myeloid differentiation primary response gene (88)
NFKB1: Nuclear factor of kappa light polypeptide gene enhancer in B-cells 1
NFKBIA: Nuclear factor of kappa light polypeptide gene enhancer in B-cells inhibitor, alpha
NF-κB: Nuclear factor of kappa light polypeptide gene enhancer in B-cells inhibitor, beta
NLRP3: NLR family, pyrin domain containing 3
NOD1: Nucleotide-binding oligomerization domain containing 1
NOD2: Nucleotide-binding oligomerization domain containing 2
RAG1: Recombination activating gene 1
RORC: RAR-related orphan receptor C
RPLP0: Ribosomal protein, large, P0
SCCmec: Staphylococcal cassette chromosome
SLC11A1: Solute carrier family 11 (proton-coupled divalent metal ion transporters), member 1
*spa*: Staphylococcal protein A
ST: Sequence types
STAT1: Signal transducer and activator of transcription 1, 91 kDa
STAT3: Signal transducer and activator of transcription 3 (acute-phase response factor)
STAT4: Signal transducer and activator of transcription 4
STAT6: Signal transducer and activator of transcription 6, interleukin-4 induced
TBX21: T-box 21
TGF-β: Transforming growth factor-beta
TGO/ALT: Alanine aminotransferase
TGO/AST: Aspartate transaminase
TI: Treated and infected group
TICAM1: Toll-like receptor adaptor molecule 1
TLR1: Toll-like receptor 1
TLR2: Toll-like receptor 2
TLR3: Toll-like receptor 3
TLR4: Toll-like receptor 4
TLR5: Toll-like receptor 5
TLR6: Toll-like receptor 6
TLR7: Toll-like receptor 7
TLR8: Toll-like receptor 8
TLR9: Toll-like receptor 9
TNF-α/TNF: Tumor necrosis factor
TRAF6: TNF receptor-associated factor 6
TYK2: Tyrosine kinase 2
WHO: World Health Organization
