# Supplementary material for: Citral modulates human monocyte responses to Staphylococcus aureus infection
Source: Sci Rep. 2021 Nov 11;11:22029. doi: 10.1038/s41598-021-01536-4 (PMC8586039; doi:10.1038/s41598-021-01536-4)
Supplement: Supplementary file 1 — Supplementary Information. [file 41598_2021_1536_MOESM1_ESM.docx]

**Supplementary material**

**Table S1.** Genes evaluated by Human Innate and Adaptive Immune Response PCR Array.

| **Symbol** | **Description** |
| --- | --- |
| APCS | Amyloid P component, serum |
| C3 | Complement component 3 |
| CASP1 | Caspase 1, apoptosis-related cysteine peptidase (interleukin 1, beta, convertase) |
| CCL2 | Chemokine (C-C motif) ligand 2 |
| CCL5 | Chemokine (C-C motif) ligand 5 |
| CCR4 | Chemokine (C-C motif) receptor 4 |
| CCR5 | Chemokine (C-C motif) receptor 5 |
| CCR6 | Chemokine (C-C motif) receptor 6 |
| CCR8 | Chemokine (C-C motif) receptor 8 |
| CD14 | CD14 molecule |
| CD4 | CD4 molecule |
| CD40 | CD40 molecule, TNF receptor superfamily member 5 |
| CD40LG | CD40 ligand |
| CD80 | CD80 molecule |
| CD86 | CD86 molecule |
| CD8A | CD8a molecule |
| CRP | C-reactive protein, pentraxin-related |
| CSF2 | Colony stimulating factor 2 (granulocyte-macrophage) |
| CXCL10 | Chemokine (C-X-C motif) ligand 10 |
| CXCR3 | Chemokine (C-X-C motif) receptor 3 |
| DDX58 | DEAD (Asp-Glu-Ala-Asp) box polypeptide 58 |
| FASLG | Fas ligand (TNF superfamily, member 6) |
| FOXP3 | Forkhead box P3 |
| GATA3 | GATA binding protein 3 |
| HLA-A | Major histocompatibility complex, class I, A |
| HLA-E | Major histocompatibility complex, class I, E |
| ICAM1 | Intercellular adhesion molecule 1 |
| IFNA1 | Interferon, alpha 1 |
| IFNAR1 | Interferon (alpha, beta and omega) receptor 1 |
| IFNB1 | Interferon, beta 1, fibroblast |
| IFNG | Interferon, gamma |
| IFNGR1 | Interferon gamma receptor 1 |
| IL10 | Interleukin 10 |
| IL13 | Interleukin 13 |
| IL17A | Interleukin 17A |
| IL18 | Interleukin 18 (interferon-gamma-inducing factor) |
| IL1A | Interleukin 1, alpha |
| IL1B | Interleukin 1, beta |
| IL1R1 | Interleukin 1 receptor, type I |
| IL2 | Interleukin 2 |
| IL23A | Interleukin 23, alpha subunit p19 |
| IL4 | Interleukin 4 |
| IL5 | Interleukin 5 (colony-stimulating factor, eosinophil) |
| IL6 | Interleukin 6 (interferon, beta 2) |
| CXCL8 | Interleukin 8 |
| IRAK1 | Interleukin-1 receptor-associated kinase 1 |
| IRF3 | Interferon regulatory factor 3 |
| IRF7 | Interferon regulatory factor 7 |
| ITGAM | Integrin, alpha M (complement component 3 receptor 3 subunit) |
| JAK2 | Janus kinase 2 |
| LY96 | Lymphocyte antigen 96 |
| LYZ | Lysozyme |
| MAPK1 | Mitogen-activated protein kinase 1 |
| MAPK8 | Mitogen-activated protein kinase 8 |
| MBL2 | Mannose-binding lectin (protein C) 2, soluble |
| MPO | Myeloperoxidase |
| MX1 | Myxovirus (influenza virus) resistance 1, interferon-inducible protein p78 (mouse) |
| MYD88 | Myeloid differentiation primary response gene (88) |
| NFKB1 | Nuclear factor of kappa light polypeptide gene enhancer in B-cells 1 |
| NFKBIA | Nuclear factor of kappa light polypeptide gene enhancer in B-cells inhibitor, alpha |
| NLRP3 | NLR family, pyrin domain containing 3 |
| NOD1 | Nucleotide-binding oligomerization domain containing 1 |
| NOD2 | Nucleotide-binding oligomerization domain containing 2 |
| RAG1 | Recombination activating gene 1 |
| RORC | RAR-related orphan receptor C |
| SLC11A1 | Solute carrier family 11 (proton-coupled divalent metal ion transporters), member 1 |
| STAT1 | Signal transducer and activator of transcription 1, 91kDa |
| STAT3 | Signal transducer and activator of transcription 3 (acute-phase response factor) |
| STAT4 | Signal transducer and activator of transcription 4 |
| STAT6 | Signal transducer and activator of transcription 6, interleukin-4 induced |
| TBX21 | T-box 21 |
| TICAM1 | Toll-like receptor adaptor molecule 1 |
| TLR1 | Toll-like receptor 1 |
| TLR2 | Toll-like receptor 2 |
| TLR3 | Toll-like receptor 3 |
| TLR4 | Toll-like receptor 4 |
| TLR5 | Toll-like receptor 5 |
| TLR6 | Toll-like receptor 6 |
| TLR7 | Toll-like receptor 7 |
| TLR8 | Toll-like receptor 8 |
| TLR9 | Toll-like receptor 9 |
| TNF | Tumor necrosis factor |
| TRAF6 | TNF receptor-associated factor 6 |
| TYK2 | Tyrosine kinase 2 |
| ACTB | Actin, beta |
| B2M | Beta-2-microglobulin |
| GAPDH | Glyceraldehyde-3-phosphate dehydrogenase |
| HPRT1 | Hypoxanthine phosphoribosyltransferase 1 |
| RPLP0 | Ribosomal protein, large, P0 |
